# Laparoscopic repeat liver resection for hepatic epithelioid hemangioendothelioma

**DOI:** 10.1186/s40792-020-01036-z

**Published:** 2020-10-01

**Authors:** Fumihiro Terasaki, Yusuke Yamamoto, Teiichi Sugiura, Yukiyasu Okamura, Takaaki Ito, Ryo Ashida, Katsuhisa Ohgi, Katsuhiko Uesaka

**Affiliations:** Division of Hepato-Biliary-Pancreatic Surgery, Shizuoka Cancer Center, 1007 Shimonagakubo, Nagaizumi-cho, Sunto-gun, Shizuoka, 411-8777 Japan

**Keywords:** Laparoscopy, Liver resection, Hepatic epithelioid hemangioendothelioma

## Abstract

**Background:**

Optimal treatment for patients with hepatic epithelioid hemangioendothelioma (HEHE) remains unclear. Laparoscopic repeat liver resection (LR) is a minimally invasive and potentially effective surgical option for multiple HEHEs.

**Case presentation:**

A 42-year-old woman with no relevant history was admitted for multiple liver tumors. Six tumors were observed on T2-weighted magnetic resonance imaging (MRI) including one in S2, two in S3, two in S7, and one in S8. Pathological evaluation of percutaneous tumor biopsy tissue suggested a diagnosis of HEHE and laparoscopic LR was planned. The procedure began with partial resection of S7 and partial resection of S8 and left lateral sectionectomy were performed. Another tumor was found intraoperatively on the surface of S6, necessitating removal by partial resection. Pathological evaluation of the resected tumor tissue from all seven tumors concurred with that of the preoperative biopsy. The patient was discharged on postoperative day 6 without any complications. A follow-up MRI 15 months after the primary surgery revealed one tumor each in S4, S6, and S8. Laparoscopic repeat LR was performed. The patient was discharged on postoperative day 5 without any complications. All three recurrent tumors were pathologically confirmed as HEHEs.

**Conclusions:**

We successfully treated primary and recurrent HEHEs with laparoscopic LR, which is a reasonable minimally invasive procedure considering the possibility of multiple courses of liver surgery in patients with HEHE.

## Background

Hepatic epithelioid hemangioendothelioma (HEHE) is a rare vascular tumor with a reported prevalence of less than one case per million [1]. Most frequently, it affects patients aged between 30 and 40 years [2]. HEHE is generally considered a less aggressive and slow-growing tumor compared to epithelioid angiosarcoma [3] and patients with HEHE often survive for over 10 years [4, 5]. However, it can rapidly progress in some patients who survive only for a year [6].

Liver resection (LR), liver transplantation (LT), and anti-cancer drugs [7–9] have been reported as effective treatments for HEHE. Both LR and LT can achieve long-term survival of patients with selected primary HEHEs, but the surgical indications and the choice of LR or LT for HEHE are still controversial due to its rarity [10]. We present a case of multiple primary HEHEs with recurrent tumors treated with laparoscopic repeat LR.

## Case presentation

**Fig. 1:**
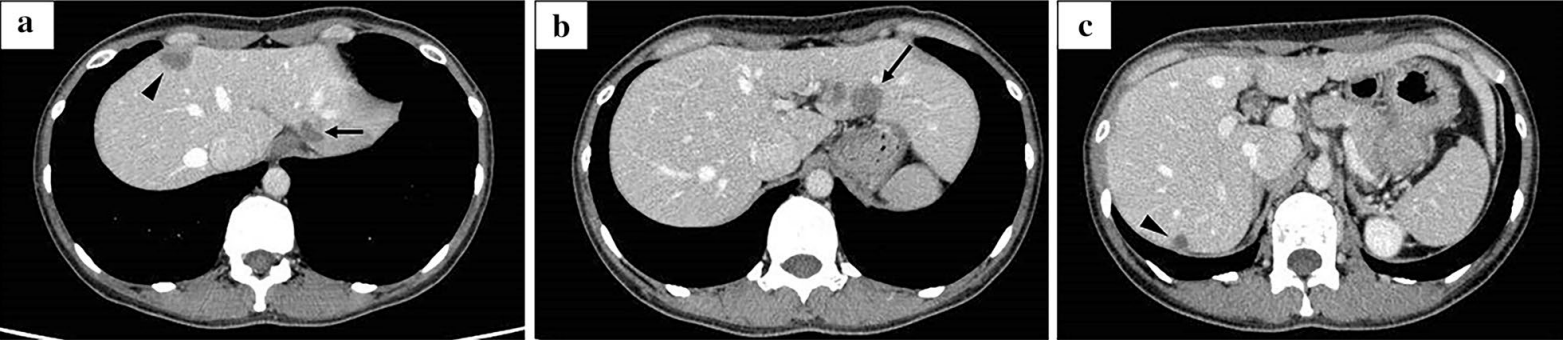
**a**–**c** Abdominal contrast-enhanced computed tomography showed four hypovascular liver tumors in the left lateral section (**a** and **b**, arrow), the posterior superior segment (S7) (**c**, arrowhead), and the anterior superior segment (S8) (Fig. 1a, arrowhead)

**Fig. 2:**
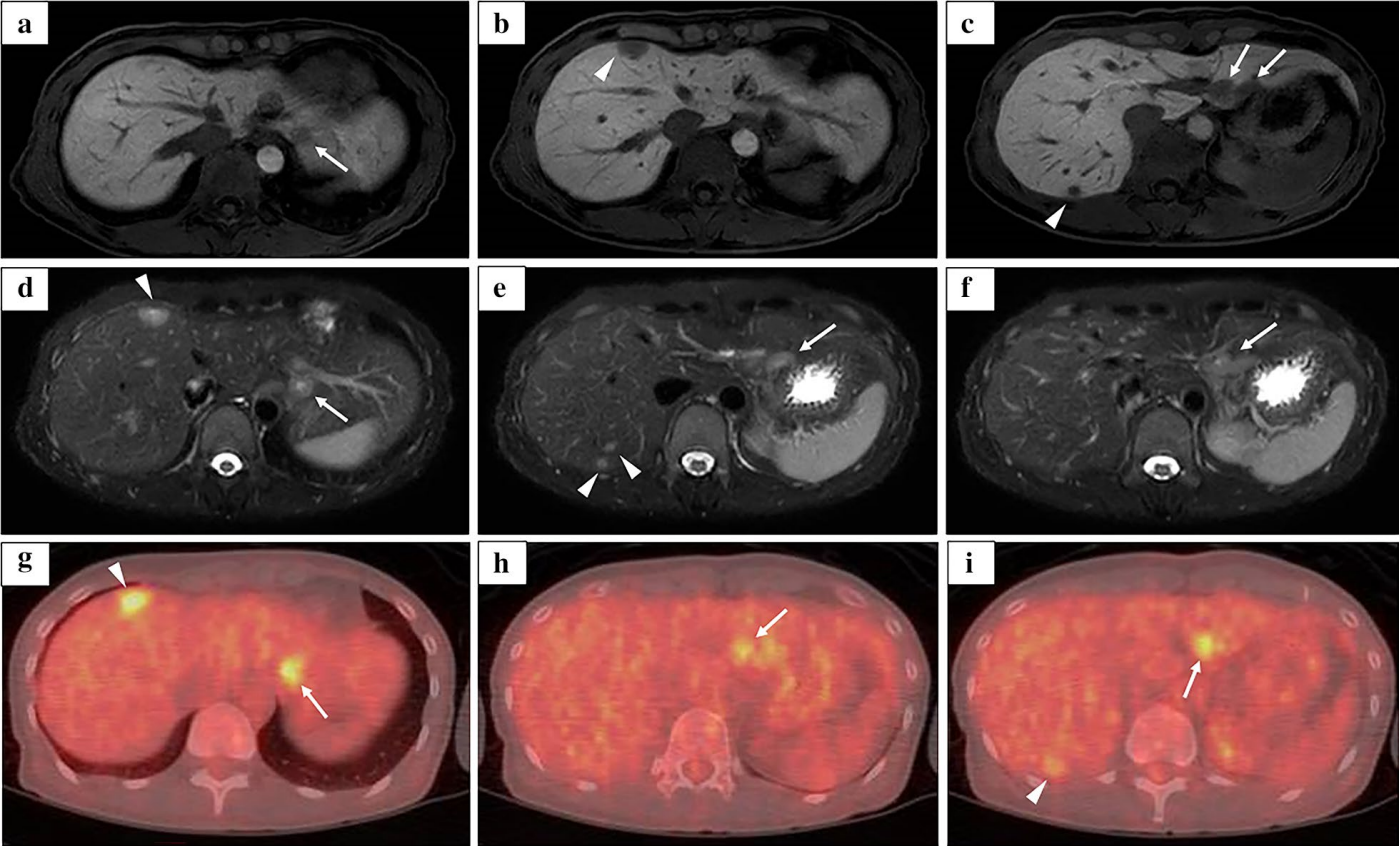
**a**–**c** Gadolinium–ethoxybenzyl–diethylenetriamine–pentaacetic acid (Gd–EOB–DTPA) enhanced magnetic resonance imaging (MRI) revealed five tumors including a 15-mm tumor in the lateral superior segment (S2) (**a**, arrow), a 20-mm tumor in the anterior superior segment (S8) (**b**, arrowhead), a 16-mm (**c**, arrow) tumor and a 20-mm tumor (**c**, arrow) in the lateral inferior segment (S3), a 10-mm tumor in the posterior superior segment (S7) (**c**, arrowhead). **d**–**f** T2-weighted MRI revealed six tumors including a 15-mm tumor in S2 (**d**, arrow), a 16-mm (**e**, arrow) tumor and a 20-mm tumor (**f**, arrow) in S3, a 3-mm tumor and a 10-mm tumor in S7 (**e**, arrowheads), and a 20-mm tumor in S8 (**d**, arrowhead). A typical target sign was observed in the tumors in S2 (**d**, arrow) and S8 (**d**, arrowhead). **g**–**i** Enhanced 2-[^18^F] fluoro-2-deoxy-d-glucose (FDG) positron emission tomography showed FDG uptake in all tumors except one 3-mm tumor in S7

**Fig. 3:**
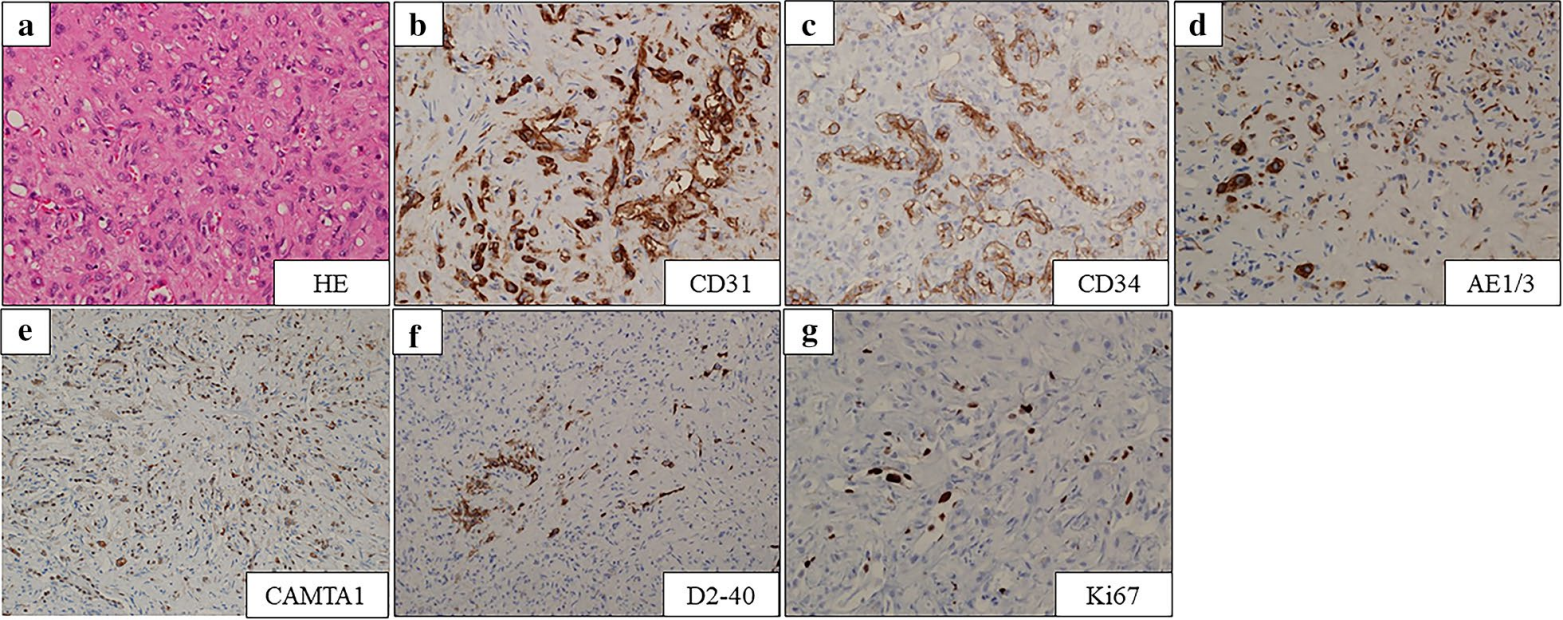
**a** Pathological evaluation of the liver biopsy showed proliferation of acidophil tumor cells surrounded by a fibromyxoid stroma (×40). **b**–**g** Immunohistochemical staining showed strong expression of CD31 (**b**, ×40), CD34 (**c**, ×40), AE1/3 (**d**, ×40), and CAMTA1 (**e**, ×20) and weak expression of D2-40 (**f**, ×20) with a Ki-67 labeling index < 10% (**g**, ×40)

A 42-year-old woman with no relevant history was admitted for the treatment of multiple liver tumors. Abdominal contrast-enhanced computed tomography (CT) revealed four hypovascular liver tumors in the left lateral section (Fig. 1a and 1b, arrow), the posterior superior segment (S7) (Fig. 1c, arrowhead), and the anterior superior segment (S8) (Fig. 1a, arrowhead). The lollipop sign [11], which is often seen in HEHE, was not observed. Gadolinium–ethoxybenzyl–diethylenetriamine–pentaacetic acid (Gd–EOB–DTPA) enhanced magnetic resonance imaging (MRI) revealed five tumors including a 15-mm tumor in the lateral superior segment (S2) (Fig. 2a, arrow), a 20-mm tumor in the anterior superior segment (S8) (Fig. 2b, arrowhead), a 16-mm (Fig. 2c, arrow) tumor and a 20-mm tumor (Fig. 2c, arrow) in the lateral inferior segment (S3), a 10-mm tumor in the posterior superior segment (S7) (Fig. 2c, arrowhead). T2-weighted magnetic resonance imaging (MRI) revealed six tumors including a 15-mm tumor in S2 (Fig. 2d, arrow), a 16-mm (Fig. 2e, arrow) tumor and a 20-mm tumor (Fig. 2f, arrow) in S3, a 3-mm tumor and a 10-mm tumor in S7 (Fig. 2e, arrowheads), and a 20-mm tumor in S8 (Fig. 2d, arrowhead). A typical target sign consisting of a core with high signal intensity, a thin ring with low-signal intensity, and a peripheral halo with slightly hyperintense signal [12] was observed in the tumors in S2 (Fig. 2d, arrow) and S8 (Fig. 2d, arrowhead). Enhanced 2-[^18^F] fluoro-2-deoxy-D-glucose (FDG) positron emission tomography (PET) revealed FDG uptake in all tumors except one 3-mm tumor (S7) (Fig. 2g–i). Pathological evaluation of percutaneous tumor biopsy tissue (Fig. 3a–g) suggested a diagnosis of HEHE, with proliferation of acidophilic tumor cells surrounded by a fibromyxoid stroma (Fig. 3a); strong immunohistochemical staining for CD31 (Fig. 3b), CD34 (Fig. 3c), AE1/3 (Fig. 3d), and CAMTA1 (Fig. 3e); and weak staining for D2-40 (Fig. 3f) with a Ki-67 labeling index < 10% (Fig. 3g). There was no evidence of metastasis on CT, PET, or gastrointestinal endoscopy. Indocyanine green (ICG) retention at 15 min (ICGR15) was 9.6%. Prognostically, the patient was classified as Child–Pugh class A. She was negative for hepatitis B virus surface antigen and anti-hepatitis C virus antibody. Her serum carbohydrate antigen 19–9 was 8 U/mL. The tumor was located in close proximity to the root of the Glissonean pedicle of S2. Laparoscopic left lateral sectionectomy and partial resection of S7 and S8 were planned. After the right lobe was mobilized, we performed the extracorporeal Pringle maneuver [13], partial resection of S7 and S8, and left lateral sectionectomy. An additional tumor was found intraoperatively on the surface of the posterior inferior segment (S6), which was removed by partial resection. INTERCEED® (Johnson & Johnson, New Brunswick, NJ, USA) was placed below the mobilized liver and umbilical port site to prevent adhesion. The operation time was 6 h and 44 min and the intraoperative blood loss was 368 g. Pathological evaluation of the specimens confirmed the diagnosis of HEHE. Surgical margin for all resected tumors was 1 mm to 10 mm. The patient was discharged on postoperative day 6 without any complications.

**Fig. 4:**
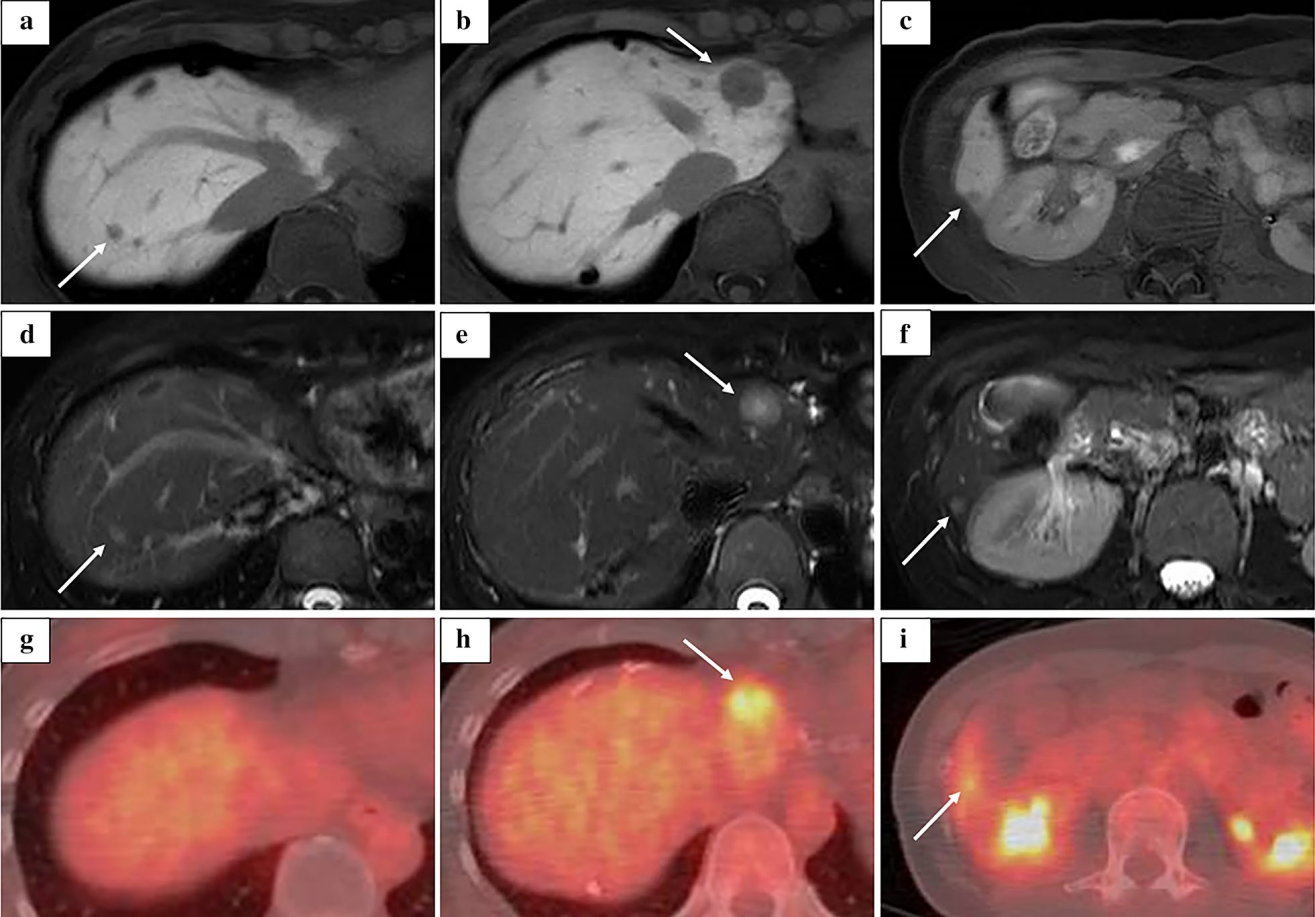
**a–f** Follow-up Gd–EOB–DTPA enhanced MRI and T2-weighted MRI at 15 months after the primary surgery revealed a 18-mm tumor in the medial section (S4) (**b**, **e**, arrow), a 7-mm tumor in the posterior inferior segment (S6) (**c**, **f**, arrow), and a 4-mm tumor in the anterior superior segment (S8) (**a** and **d**, arrow). **g**–**i** Enhanced FDG-PET showed uptake of FDG in the tumor in S4 and S6 (**h**, **i**)

**Fig. 5:**
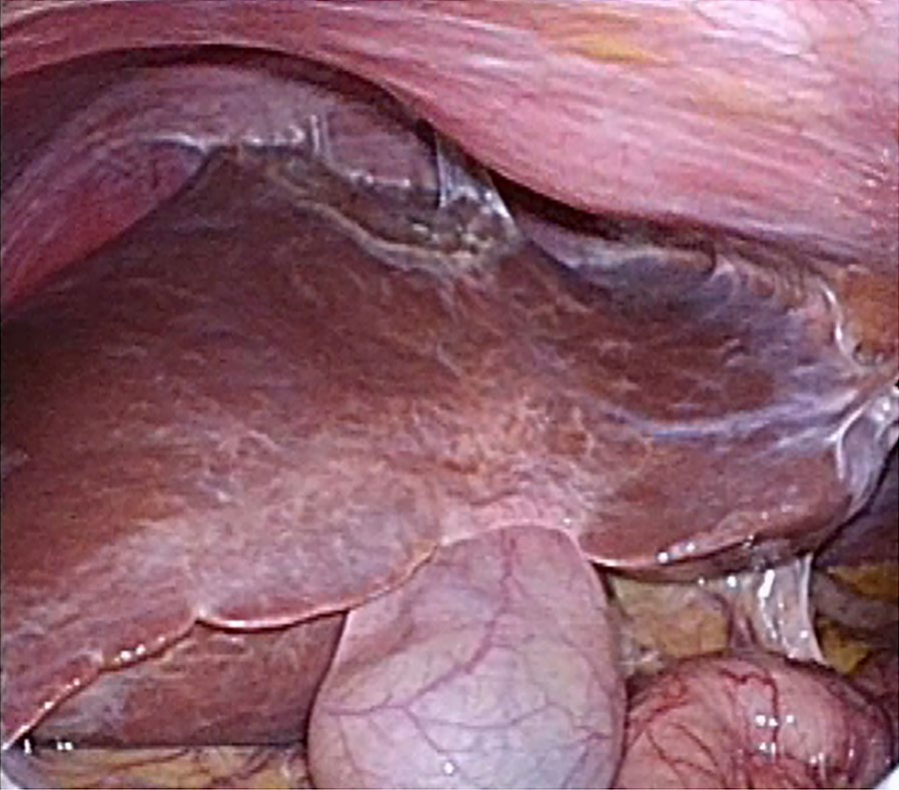
Absence of severe adhesions in laparoscopic repeat LR

**Fig. 6:**
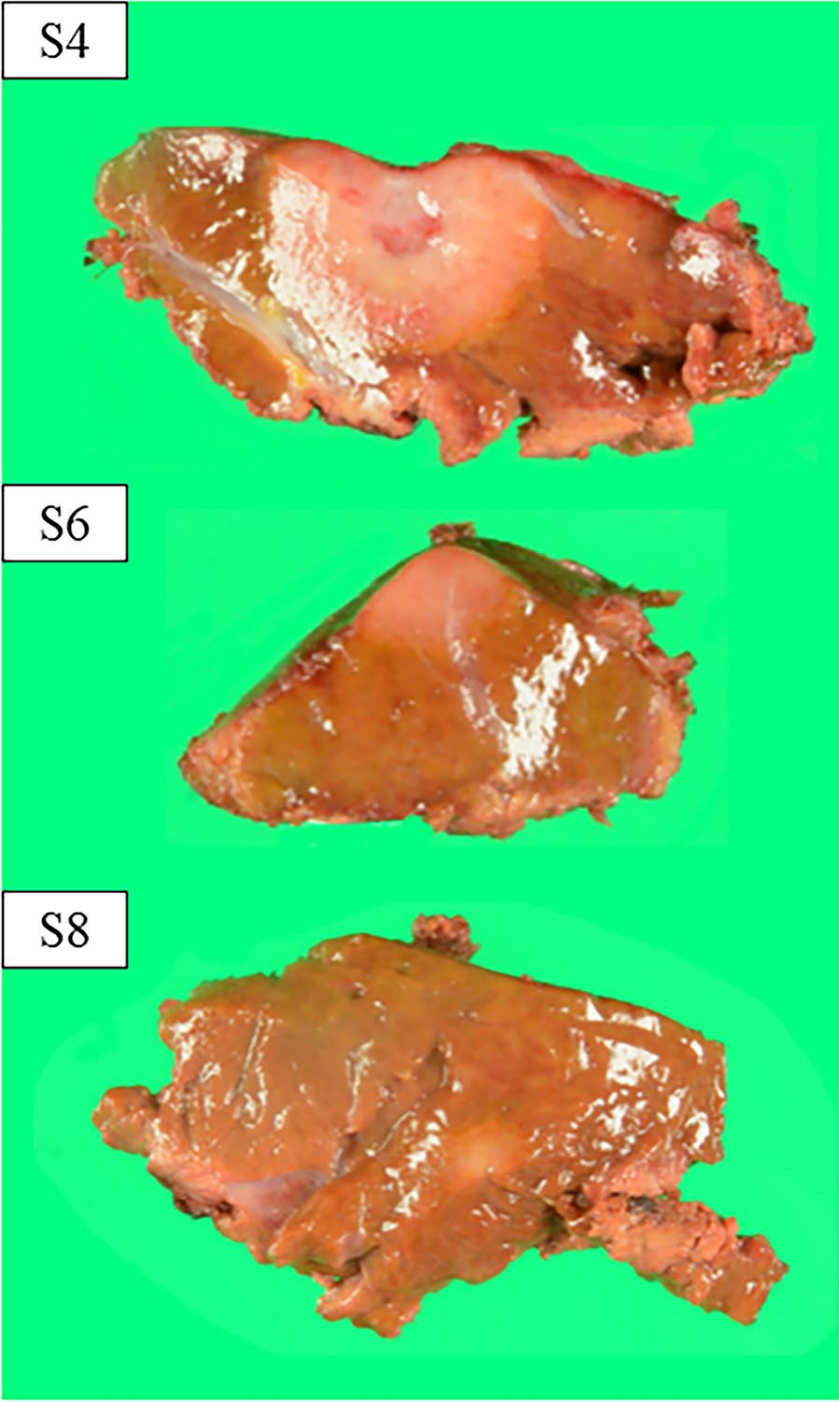
Surgical margin for all resected tumors was 3 mm to 10 mm

Follow-up Gd–EOB–DTPA-enhanced MRI and T2-weighted MRI, performed 15 months after the primary surgery, revealed an 18-mm tumor in the medial section (S4) (Fig. 4b, e, arrow), 7-mm tumor in S6 (Fig. 4c, f, arrow), and 4-mm tumor in S8 (Fig. 4a, d, arrow). Enhanced FDG-PET showed uptake of FDG in the tumor in S4 and S6 (Figs. 4h, i). The ICGR15 was 10.0%. Laparoscopic LR was planned at 2 years and 3 months after the primary surgery due to the patient’s wishes and the slow growth of the tumors. Preoperative contrast-enhanced ultrasonography was performed using perfluorobutane (Sonazoid®), which only revealed the tumor in S4 and failed to detect the tumors in S6 and S8. We injected ICG into the patient according to methods described in a previous report [14]. In the second surgery, no severe adhesions were noted (Fig. 5), and the extracorporeal Pringle maneuver was easily performed. All tumors were identified on intraoperative ultrasonography; however, the tumors in S6 and S8 could not be detected by fusion-fluorescence imaging using ICG. Laparoscopic partial LR of S4, S6, and S8 was performed. The operation time was 7 h and 8 min with an intraoperative blood loss of 300 g. Blood transfusion was avoided. The patient was discharged on postoperative day 5 without any complications. The pathological evaluation of all three tumors confirmed the diagnosis of HEHE. Surgical margin for all resected tumors was 3 mm to 10 mm (Fig. 6). Follow-up MRI 3 months after the second surgery revealed no evidence of recurrence.

## Discussion

The present case report is the first one describing the use of laparoscopic repeat LR in a patient with HEHE. HEHE is considered a borderline neoplastic tumor, which is generally slow-growing. However, some HEHEs are rapidly progressing tumors similar to angiosarcomas. Treatments for HEHE include LR, LT, chemoradiotherapy, and observation and the reported 5-year overall survival rates of these therapies were 75%, 54.5%, 30%, and 4.5%, respectively [15]. In the largest review of literature [15], 45.7% of the patients presented with tumor recurrence regardless of the treatment modality and the most common recurrence site was the liver. Thus, HEHE has a certain possibility of recurrence in the liver. Repeat LR may contribute to longer survival and appropriate procedure should be chosen at the time of the initial appearance of HEHE, taking into account the multiple courses of liver surgery.

Both LR and LT are considered effective treatments with acceptable long-term survival [16]. However, LT should be chosen over LR only after careful consideration of post-transplant mortality and morbidity such as infection [17, 18] and graft failure [19]. LT is associated with greater blood loss, longer operating procedures, and longer hospital stay [20] when compared with LR. For patients receiving LT, the reported early (≤ 3 months) and late (> 3 months) mortality rates were 1% –5% and 22% [15, 21], respectively, which were higher than the mortality rates of LR (0% –1%) [22, 23]. In a previous case, multiple bone and spleen metastases developed within 3 months after LT [3], suggesting the possibility of occasional aggressive tumor recurrence after LT. Thus, even LT cannot always prevent the recurrence of HEHE.

The advantages of laparoscopic LR for HEHE have not been well documented. However, it is a reasonable procedure considering the features of HEHE. HEHE often appears in a form of multiple tumors near the surface of the liver [24], making partial LR possible. Laparoscopic LR involves little intraoperative blood loss and requires a short postoperative hospital stay. It is associated with limited postsurgical tissue adhesion [25] and is suitable for the nature of HEHE, which sometimes requires repeat LR for intrahepatic recurrence.

Laparoscopic repeat LR involves less intraoperative blood loss and shorter hospital stay than open repeat LR when a patient’s liver function is favorable and the tumors are small [26, 27]. Patients with HEHE meet the conditions of these features, and laparoscopic repeat LR should be recommended for patients with recurrence of HEHE.

In the present case, all tumors were located near the liver surface, and adhesion was limited in the second surgical procedure despite the mobilization of the right lobe in the primary LR. The Pringle maneuver was easily performed, and the tumors were safely resected. Our criteria for conversion to laparotomy—estimated blood loss > 2000 mL, long operation time, difficulty in adhesiolysis, and difficulty in repairing major vessels or organs—did not require conversion to laparotomy. Thus, laparoscopic repeat LR was considered a beneficial choice for the treatment of HEHE.

The appropriate surgical margin for HEHE remains controversial. A previous study demonstrated that tumor cells are occasionally observed in sinusoids or central veins within 10 mm from the main HEHE; however, this did not correlate with recurrence and prognosis, and none of the patients with surgical margins < 1 mm had recurrence [28]. It has also been reported that negative surgical margins are associated with improved survival in all liver sarcomas, including HEHE [10]. In the present case, the surgical margin was 1–10 mm in the primary surgery and 3–10 mm in the second surgery. Local recurrence was not observed. These results indicate that surgical margins > 10 mm are not required, and resection for HEHE should be performed with a negative surgical margin.

In summary, laparoscopic LR is a reasonable procedure considering the possibility of multiple courses of liver surgery in HEHE patients, as this procedure is minimally invasive. It results in relatively low morbidity and mortality and limited adhesion. Appropriate surgical margins are essential while performing laparoscopic LR for HEHE.

Diagnostic imaging for HEHE is sometimes difficult to interpret due to the nature of tumors. On MRI, the most frequent presentation is generally a peripheral distribution of the lesions and target appearance on T2-weighted images [29]. Gd–EOB–DTPA-enhanced MRI, which reveals hypointense lesions, is also useful [30]. On PET, FDG uptake was observed only in 66% of patients with HEHE in a previous study [31]. During the primary surgery in the present case, we detected 6 tumors on T2-weighted images, whereas Gd–EOB–DTPA-enhanced MRI and PET revealed 5 tumors, and the 3-mm tumor in S7 was not detected using these modalities. During the second surgery, both T2-weighted images and Gd–EOB–DTPA-enhanced MRI revealed 3 tumors, whereas PET revealed 2 tumors and could not detect the 4-mm tumor in S8. The components of all the resected tumors were the same and involved a fibrous stromal area at the center of the lesion with tumor cells surrounding the fibrous area. The sensitivity of each modality may have been affected if the tumors were too small.

## Conclusions

We successfully treated primary and recurrent HEHE with laparoscopic LR. Laparoscopic LR is minimally invasive and with appropriate patient selection, and it can be performed repeatedly in patients with intrahepatic tumor recurrence.

## Data Availability

Datasets supporting the conclusions of this article are included within the article.
